# Primary Humoral Immunodeficiencies and Bronchiectasis in Adults

**DOI:** 10.3390/jcm15010179

**Published:** 2025-12-26

**Authors:** Guillermo Suárez-Cuartín, Carmen Lores, Jose Daniel Gomez-Olivas, Grace Oscullo, Miguel Ángel Martínez-García

**Affiliations:** 1 Pneumology Department, Bellvitge University Hospital, Bellvitge Biomedical Research Institute (IDIBELL), University of Barcelona, 08907 Barcelona, Spain; clores@bellvitgehospital.cat; 2 Centro de Investigación Biomédica en Red de Enfermedades Respiratorias, Instituto de Salud Carlos III, 28029 Madrid, Spain; jose_gomez@iislafe.es (J.D.G.-O.); graceoscullo@gmail.com (G.O.); mianmartinezgarcia@gmail.com (M.Á.M.-G.); 3 Pneumology Department, Health Investigation Institute La Fe (IISLAFE), La Fe Polytechnic and University Valencia, 46026 Valencia, Spain

**Keywords:** immunodeficiency, bronchiectasis, immunoglobulins

## Abstract

Primary humoral immunodeficiencies are a heterogeneous group of disorders defined by quantitative and/or functional defects in one or more immunoglobulin classes, often with associated cellular immune abnormalities. Their link with bronchiectasis, whose prevalence varies across specific defects, is largely driven by recurrent respiratory infections. Selective Immunoglobulin-(Ig)A deficiency and IgG2 subclass deficiency are the most frequent forms, but common variable immunodeficiency (CVID) is the condition most often associated with bronchiectasis and is usually diagnosed earlier because of its characteristic phenotype. In contrast, the contribution of isolated IgA deficiency or selective IgG subclass deficiencies to bronchiectasis remains controversial. Other reported associations include X-linked agammaglobulinemia, selective IgM or IgG deficiency, and rarer entities such as selective IgE deficiency, unclassified hypogammaglobulinemia, specific antibody deficiency, specific polysaccharide antibody deficiency, and heavy- or light-chain deficiencies. Current bronchiectasis guidelines recommend measurement of serum immunoglobulins and IgG subclasses in patients with compatible features, recurrent infections, or no clear etiology before labeling disease as idiopathic. Identifying immunoglobulin defects is clinically important because they represent treatable traits. The potential role of emerging therapies such as the DPP1 inhibitor brensocatib in immunodeficiency-related bronchiectasis remains uncertain, and ongoing registries will be key to clarifying these relationships.

## 1. Introduction

Primary humoral immunodeficiencies (PHI) constitute a heterogeneous group of immune disorders characterized by a quantitative deficiency or functional impairment of antibodies (immunoglobulins). This defect affects the respiratory system in various ways, depending on the specific disorder and other contributing factors [1].

Various organizations maintain registries of immunodeficiencies, including humoral types, such as ESID (European Society for Immunodeficiencies) [2], USIDNET (US Immunodeficiency Network Registry) [3], and IUIS (International Union of Immunological Societies), which defines, classifies, and standardizes the nomenclature and diagnostic criteria of these diseases [4]. Numerous smaller national or regional registries also exist. The IUIS classifies humoral immunodeficiencies into four main groups, shown in Table 1 [4,5].

The prevalence of PHI depends on patient age and the specific type of immunodeficiency. It is estimated that 1 in 1000–2000 individuals in the general population are affected, with lower prevalence in adults (1 in 25,000–50,000). Diagnostic delays are common due to low clinical suspicion, subclinical or late-onset symptoms, or misattribution of symptoms to chronic diseases, medications, or procedures [1].

Immunoglobulin-(Ig)A deficiency is the most common PHI, affecting 1 in 200–500 individuals [6], followed by common variable immunodeficiency (CVID) (1 in 25,000–50,000) [7], IgG subclasses deficiency (1 in 500–2000) [8], and IgM deficiency (1 in 100,000–200,000) [9]. A reduced immunoglobulin production or function compromises immunity, particularly against encapsulated bacteria [1].

Abnormal immunoglobulin values are generally those that fall outside the mean ±2 standard deviations of the general population [10]. Normal adult immunoglobulin ranges are: IgA: 70–400 mg/dL (0.7–4 g/L); IgM: 40–230 mg/dL (0.4–2.3 g/L); IgG: 700–1600 mg/dL (7–16 g/L); IgG subclasses: (IgG1: 430–1050 mg/dL; IgG2: 250–650 mg/dL; IgG3: 50–200 mg/dL and IgG4: 30–150 mg/dL) [10].

IgG is the most abundant immunoglobulin, with IgG1–IgG4 representing ~60–70%, 20–30%, 5–8%, and 1–4% of total IgG, respectively [11]. Although isolated IgG subclass deficiencies are rare (<1%), combined deficiencies are even less common. These deficiencies can increase susceptibility to respiratory and upper airway infections, particularly those caused by encapsulated bacteria. Table 2 summarizes the main role and consequence of the deficiency of the different immunoglobulins [12,13,14].

Certain immunodeficiencies, such as CVID and X-linked agammaglobulinemia (Bruton’s disease), involve both humoral and cellular components and are associated with severe infections from early childhood. CVID, for example, is characterized by poor response to polysaccharide vaccines (e.g., *Haemophilus influenzae* and *Streptococcus pneumoniae*) [6,15].

This narrative review focuses on the relationship between common PHI and the development of non-cystic fibrosis bronchiectasis (hereafter referred to as bronchiectasis).

## 2. Methods

In general, the existing scientific evidence regarding the association between different types of humoral immunodeficiencies and bronchiectasis is very limited. A systematic literature review was conducted using the PubMed and Embase databases, employing the following terms in the title: (Immunodeficiency OR IgA OR IgA1 OR IgA2 OR IgG OR IgM OR IgG subclasses OR IgG1 OR IgG2 OR IgG3 OR IgG4 OR Combined immunoglobulin OR Common variable immunodeficiency OR CVID OR immunoglobulin OR humoral immunodeficiency OR agammaglobulinemia OR Bruton)* AND in the title or abstract: (Bronchiectasis OR bronchial infection). Regarding clinical studies, only those conducted in adult populations were included in the analysis (PubMed filter: Adults 19+ years).

A total of 138 manuscripts were identified. Two authors of the present study (GSC and MAMG) independently screened the manuscripts, and any discrepancies were resolved by consensus. The majority of the identified papers were case reports or referred to the detection of *Aspergillus* sp. or *Pseudomonas aeruginosa* infections through immunoglobulin assays.

After the screening process, 45 manuscripts were included in the final review. Among these, the most frequently studied humoral immunodeficiencies were isolated or combined IgG subclass deficiencies (16 manuscripts), CVID (10), IgA deficiency (4), and agammaglobulinemia (4). Fewer than four manuscripts addressed IgG deficiency, combined immune defects, and hyper-IgM syndrome. Additionally, five manuscripts discussed immunoglobulin replacement therapy in relation to these syndromes.

Of all the manuscripts reviewed, the term “bronchiectasis” appeared explicitly in 11 papers, usually in reference to IgG subclass deficiencies.

## 3. Humoral Immunodeficiencies and Bronchiectasis

Bronchiectasis refers to dilations—typically irreversible—of the bronchial tree, which are commonly accompanied by bronchial wall thickening as an indirect marker of inflammation [16]. This inflammation, to varying degrees, impairs local defense mechanisms such as the mucociliary system, increasing susceptibility to infections by pathogenic bacteria. These infections, in turn, further exacerbate bronchial inflammation, creating a vicious cycle of infection and inflammation that drives the progression of the disease. Most of the currently available pharmacological treatments are aimed at interrupting this cycle [17].

An important point is that, according to a recent global consensus, a true diagnosis of bronchiectasis refers to radiological images of bronchial dilatation accompanied by compatible symptoms—such as chronic productive cough with a purulent component and a history of infective-type exacerbations, and not imaging alone [16].

Bronchiectasis can be caused by more than one hundred different pulmonary and extrapulmonary conditions. Among the most frequent etiologies are post-infectious bronchiectasis (including post-tuberculosis forms), as well as those associated with chronic obstructive pulmonary disease (COPD) and asthma [18]. Beyond these common causes, there are dozens of other etiologies, each accounting for a smaller proportion of cases (typically less than 5%). These fewer common etiologies may nonetheless present with distinctive clinical features [19].

A diagnosis of idiopathic bronchiectasis—or more accurately, bronchiectasis of unknown etiology—should only be made after a thorough clinical history is obtained and all appropriate complementary investigations have been performed and returned negative [20,21]. In this regard, current international guidelines for the diagnosis and management of bronchiectasis recommend serum immunoglobulin evaluation in all patients, in order to rule out humoral immunodeficiency [19,22,23,24].

Among the well-established causes of bronchiectasis are immunodeficiencies, particularly humoral immunodeficiencies [21]. Table 3 presents the proportion of immunodeficiencies among all patients with bronchiectasis, as reported in national and international registries, as well as in large-scale studies focused on the etiology of bronchiectasis. In most cases, these studies do not distinguish between humoral and cellular immunodeficiencies, grouping them under the broader term of primary immunodeficiencies. Based on the total number of patients included in these studies and the partial percentages reported, it can be estimated that approximately 4–5% of bronchiectasis cases are attributable to primary immunodeficiencies [25]. Only Martínez-García et al. differentiated PHI from the rest (secondary and cellular immunodeficiencies), observing that the vast majority (4.2% vs. 0.5%) were PHI, from which we can infer that the same is likely to occur in most of the other studies [25,26].

The reported prevalence of PHI in large bronchiectasis cohorts varies considerably, ranging from 9% in the study by Olveira et al. [26] to less than 1% in data from India [27]. In the United States, the prevalence is approximately 5% [28], while in Europe it is similar overall (4.1%), though with notable geographic variation [29]. For example, in southern Europe, the prevalence is less than half that reported in south-eastern and western Europe (Table 3). On the other hand, a cohort from Colombia including 161 patients, showed that immune disorders were among the main causes of bronchiectasis, represented by autoimmunity (13.6%) and immunodeficiency (11.7%), especially in individuals under 50 years of age [30].

Several factors may contribute to these differences. The most likely explanation is under diagnosis, due to low clinical suspicion and the limited use of appropriate diagnostic tests, which results in an overall reduction in identified cases. Additionally, many studies do not differentiate between humoral, cellular, and secondary immunodeficiencies, which affects the accuracy of reported percentages. Genetic, socioeconomic, and environmental factors may also play a role. Humoral immunodeficiencies are frequently caused by genetic defects or mutations, which may be more prevalent in regions with higher rates of consanguinity, where cultural practices promote marriage among close relatives [21]. Finally, some studies may include a disproportionately high number of patients from immunodeficiency referral centers, artificially inflating the relative percentage of immunodeficiency-related bronchiectasis. This phenomenon is clearly illustrated in the comparison between two Spanish registries. In the earlier registry [26], a single center contributed over 200 patients with immunodeficiencies, resulting in a relatively high prevalence of over 9%. In contrast, the more recent registry which did not include data from that center, reported a prevalence of immunodeficiencies less than half that of the previous study [25].

**Figure 1:**
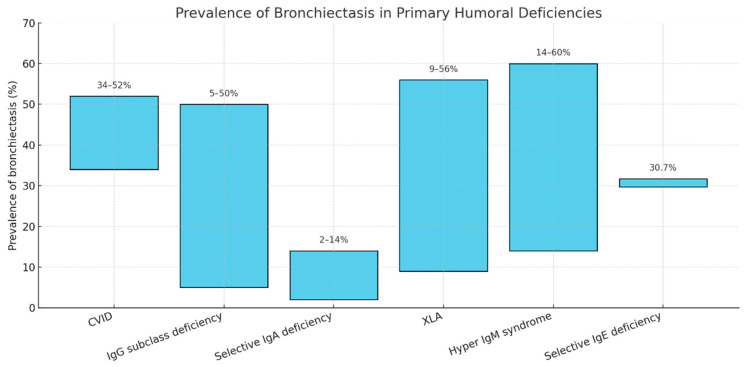
Prevalence ranges of bronchiectasis according to the different primary humoral immunoglobulin deficiencies. CVID: Common Variable Immunodeficiency; X-linked Agammaglobulinemia.

The most frequent PHIs will be described below. The estimated prevalence of bronchiectasis among them is shown in Figure 1.

Combined immunodeficiencies with syndromic features, such as STAT3-related hyper-IgE syndrome and DOCK8 deficiency, may also result in bronchiectasis [5,31]; however, as they are not classified as predominantly antibody deficiencies, they lie outside the scope of this review and will not be discussed further.

**Table 3:** Prevalence of primary humoral immunodeficiencies in large series of bronchiectasis series.

Study	Country	Year	n°	ID%
Lonni et al. [32]	6 European countries	2015	1258	5.8%
Aksamit et al. [28]	US	2017	1775	5%
Olveira et al. [26]	Spain	2017	2047	9.4%
Henkle et al. [33]	US Medicare	2018	175,572	3.5% *
Dhar et al. [27]	India	2019	2195	<1%
Visser et al. [34]	Australia	2019	566	3.7%
Huang et al. [35]	Taiwan	2020	15,729	1.3%
Martínez-Garcia et al. [25]	Spain	2021	1912	4.2%
Yu et al. [36]	South Korea	2022	931	2.8 *
Chalmers et al. [29]	Europe	2023	16,963	4.1%
-UK	--	--	8163	2.5%
-North/West	--	--	4295	6%
-South	--	--	3444	6.1%
-Central/East	--	--	1061	2.7%
Edis et al. [37]	Turkey	2024	1035	1.3%
Ibrahim et al. [38]	Qatar	2024	284	3.5%
Zea-Vera et al. [30]	Colombia	2024	161	11.7%
Burgel et al. [39]	France	2025	630	3.5%
Xu et al. [40]	China	2025	9501	0.3%

ID: Immunodeficiency; US: United States; UK: United Kingdom. * Also includes allergic bronchopulmonary aspergillosis, primary ciliary dyskinesia and others.

### 3.1. IgG Subclass Deficiencies

Although the existing literature remains limited, the thresholds of normality for defining an IgG subclass deficiency are established when one or more subclass levels are found to be two standard deviations below the age-adjusted range in patients with normal total serum IgG levels [10,11]. A persistent controversy remains regarding whether certain isolated or combined subclass deficiencies may in fact cause bronchiectasis as a consequence of recurrent respiratory infections associated with such deficiencies. The classic data supporting this notion date back to the 1990s and the beginning of the 21st century. Popa et al. reported that among 42 adults with some degree of IgG subclass deficiency and multiple acute bronchial exacerbations, there was a greater degree of chronic airflow obstruction [41]. In turn, Hill et al. proposed that, owing to the lack of consensus in defining IgG subclass deficiencies, true plasma deficits are rare, and that the pulmonary manifestations are more likely to reflect bronchial rather than systemic subclass deficiencies, which they assessed through measurement of subclass concentrations in sputum [42].

Two studies from a Spanish research group further elucidated this possible association. Thus, De Gracia et al. observed, in a cohort of 65 patients, that within the subgroup of individuals with bronchiectasis of unknown origin (defined as those cases remaining without an identified etiology despite an exhaustive diagnostic workup), thirty-one patients (48%) exhibited low serum concentrations of one or more IgG subclasses (19 IgG2 deficiencies, 2 IgG3 deficiencies, 3 IgG4 deficiencies, and 7 combined subclass deficiencies). Although this finding did not establish causality, it did highlight that the prevalence of IgG subclass deficiency was greater in this group of idiopathic bronchiectasis than in the general population, thereby justifying the inclusion of subclass determination in the diagnostic evaluation prior to classifying bronchiectasis as idiopathic [43].

Rodrigo et al. advanced this line of investigation with a study of 107 patients with bronchiectasis, hypothesizing that even in the presence of normal total IgG or subclass concentrations, a functional deficit might exist. To test this hypothesis, they evaluated the antibody response to a pneumococcal unconjugated vaccine and a *Haemophilus influenzae* type b conjugate vaccine. They observed a deficient response in 11% of patients, particularly among those with reduced IgG2 levels, suggesting that IgG2 deficiency or dysfunction may be the most relevant in relation to bronchiectasis. This notion is supported by the known role of IgG2 in the immune response to capsular bacterial polysaccharides, precisely those most frequently implicated in bronchiectasis (*Pseudomonas aeruginosa* and *Haemophilus influenzae*) [44]. Similar results were obtained by Martínez-García et al. in a cohort of 128 patients with idiopathic bronchiectasis, with a mean age of 71.6 years, where 12.5% presented at least one subclass deficiency, the most frequent being IgG2 [45].

By contrast, other studies, such as that of Drogu et al., reported that the presence of bronchiectasis in cases of selective IgG subclass deficiency is an uncommon finding in adults over 18 years. In their retrospective series of 270 patients, 96 had an IgG subclass deficiency, yet bronchiectasis was present in only 19 of them (10.4%), in comparison with other humoral immunodeficiencies. However, in the subgroup with combined IgG2 + IgG4 deficiency, prevalence was higher (30.8%), although the number of patients was very small [46].

In 2022, Zhang et al. confirmed prior observations by demonstrating that IgG2 deficiency—the most frequent subclass deficiency—was the one most closely associated with the development of bronchiectasis. Serum IgG2 levels were stratified into tertiles (<2.68 g/L, 2.68–3.53 g/L, and 3.54–4.45 g/L). Independent predictors of three or more exacerbations included: hospital admission within the preceding two years, colonization with potentially pathogenic organisms, asthma, and reduced IgG2 levels. Patients in the lower two tertiles (<2.68 g/L and 2.68–3.53 g/L) experienced a worse progression of bronchiectasis, as assessed by the Bronchiectasis Severity Index over one year, compared with those who were normal IgG2 (>4.45 g/L) (*p* = 0.013) [47].

It is therefore of paramount importance to recognize, in patients with recurrent infections and bronchiectasis, that either quantitative or functional IgG subclass deficiency (particularly of IgG2) may be associated with the presence of bronchiectasis, even when total IgG levels are within the normal range, before labeling the disease as idiopathic. Furthermore, and of greatest clinical relevance, is the fact that IgG or IgG subclass deficiencies represent a “treatable trait,” since the use of intravenous or subcutaneous immunoglobulin replacement therapy may lead to a clinically meaningful reduction in respiratory infections and prevent progressive decline in lung function [48].

### 3.2. Common Variable Immunodeficiency (CVID)

CVID are the most frequent symptomatic primary immunodeficiencies diagnosed in adulthood, and presents as a heterogeneous condition characterized by hypogammaglobulinemia (especially IgG, IgA and/or IgM), poor antibody response to vaccinations and recurrent upper and/or lower airway infections, as well as several other systemic manifestations, ranging from autoimmunity to lymphoproliferative disorders [49,50]. One of the most common features of CVID is their respiratory system involvement, which can present as bronchiectasis related to the infection-inflammation-tissue damage vicious cycle, or as immune-mediated interstitial lung disease [51]. Different studies have estimated that bronchiectasis is present in around 34–52% of patients with idiopathic hypogammaglobulinemia and CVID [52,53,54,55,56], although in some cohorts, pulmonary lymphoproliferative granulomatous disease are the main causes of morbidity and mortality in patients with CVID [56]. On the other hand, immunodeficiencies account for the main cause of bronchiectasis in approximately 1–9.4% of the cases in different bronchiectasis registries [18]. These registries, however, often do not differentiate between the specific conditions causing the immunodeficiency, so the true prevalence of CVID among bronchiectasis patients is hard to establish.

A study by Buso et al. observed that patients with CVID and bronchiectasis have a significantly lower forced expiratory volume in the first second (FEV_1_) compared to those without bronchiectasis [53]. This finding was also observed in another study by Sperlich et al., where the authors identified a more rapid FEV_1_ decline in CVID individuals with bronchiectasis [54]. Furthermore, the presence of bronchiectasis in CVID was associated with a higher rate of annual respiratory infections and worse quality of life [54]. On the other hand, this same study observed that lower serum IgM levels were associated with a higher risk of developing bronchiectasis and a higher number of annual respiratory infections [54]. In this regard, a meta-analysis by Ramzi et al. also observed that CVID patients with bronchiectasis had significantly lower levels of serum IgA and IgM, and a higher frequency of pneumonias, sinus infections, otitis media and lymphocytic interstitial pneumonia [55]. Similar results were observed in an analysis of 1470 CVID patients included in the USIDNET registry, were lower serum IgA levels, chronic rhino sinusitis, pneumonia, chronic obstructive pulmonary disease (COPD) and interstitial lung disease (ILD) were independently associated with the development of bronchiectasis [57]. In summary, these findings highlight the significant negative clinical impact of the association of CVID and bronchiectasis.

Although, in general, it was considered that the presence of bronchiectasis did not impact mortality in CVID patients, a more recent study showed that higher bronchiectasis severity score at diagnosis, as well as chronic bronchial infection by Pseudomonas aeruginosa, low FEV_1_ and the overlap with COPD do have an independent association with an increased mortality [58]. Moreover, a specific analysis of a New Zealand CVID cohort showed that early-onset disease and delayed diagnosis was associated with an increased risk of bronchiectasis as well as with premature death [59].

Immunoglobulin reposition is one of the main pillars of CVID treatment, which can help to obtain respiratory infections control. Due to this specific treatment option, the measurement of serum immunoglobulins is recommended in all bronchiectasis patients, as mentioned previously [19,22]. The efficacy of this treatment in subjects with CVID, however, may be influenced by the presence of bronchiectasis, as the required reposition dose is higher and time to achieve target IgG levels, and therefore, time to infection frequency control, takes longer in this subgroup of patients [60,61]. Nevertheless, a study by Pereira et al. observed that immunoglobulin treatment in patients with CVID and bronchiectasis managed to significantly improve airway inflammatory markers, as well as the mucus transportability by cough [62]. Another key treatment in patients with bronchiectasis is daily airway clearance through physiotherapy. In this regard, a study by Thickett et al. observed that despite the high prevalence of bronchiectasis among a cohort of CVID patients, only few patients had received instructions in airway clearance techniques [63]. These differences manifest the need to increase awareness efforts for early diagnosis and treatment of both CVID and bronchiectasis among clinicians.

### 3.3. IgA Deficiency

Selective IgA deficiency is the most common immunodeficiency [12]. Both IgA1 and IgA2 subclasses increase locally in the presence of local inflammation/infection or purulent sputum, particularly IgA2 [12,64]. Subjects with IgA deficiency may remain asymptomatic or present with bacterial infections (mainly due to encapsulated bacteria such as Streptococcus pneumoniae and Haemophilus influenzae) as well as non-bacterial infections of the respiratory tract and upper airways. IgA deficiency is also associated with certain autoimmune diseases and is frequently combined with other humoral deficiencies [65].

Regarding its relationship with bronchiectasis, results remain heterogeneous. For instance, Hodkinson et al., in a study of 626 patients with primary immunodeficiency, observed that 48% of those with combined IgA and IgM deficiency developed bronchiectasis, even though a high proportion of them were already receiving IgG replacement therapy. Among the limitations of this study was the inclusion of all types of humoral immunodeficiencies, such as CVID and X-linked agammaglobulinemia [66].

In contrast, Aghamohammadi et al. studied 37 patients with IgA deficiency and found that only 4 developed bronchiectasis, although many displayed atopic disorders. In 50% of cases, IgA deficiency was associated with other humoral deficits, particularly IgG subclass deficiency, whether quantitative or functional. Patients with this combined defect experienced a higher frequency of bronchial infections and bronchiectasis [67].

On the other hand, Domingue et al. evaluated 330 patients with selective IgA deficiency and found that although a high proportion suffered from recurrent respiratory tract infections, atopy, and certain autoimmune diseases, bronchiectasis was documented in only 6 patients. This very small percentage is likely explained by the fact that only patients younger than 18 years were included, meaning that recurrent respiratory tract infections had not yet had sufficient time to induce the development of bronchiectasis [68].

At present, no replacement therapy exists for IgA deficiency. However, several animal model trials have yielded promising results. Vonarburg et al., using a murine model, demonstrated the feasibility of topically applying human plasma-derived immunoglobulins into the lungs through a nebulized liquid formulation, which successfully prevented acute respiratory infection [69].

### 3.4. X-Linked Agammaglobulinemia (XLA)

XLA is a primary immunodeficiency caused by different mutations in the gene encoding Bruton’s tyrosine kinase (BTK), which alter B-cell development. Since the BTK gene is located on the X chromosome, the disease almost exclusively affects males [70]. Agammaglobulinemia is defined by recurrent infections before 5 years of age, IgG serum levels <500 mg/dL, IgA and IgM levels <2 standard deviations below the mean for age, and <2% circulating B cells [71].

XLA is rare, with a prevalence of 0.3 per 100,000 male births in the United Kingdom, representing 4% of patients in the UK Primary Immunodeficiency Registry [72]. Symptoms usually begin in infancy, with a median onset age of 8–12 months, and a median age at diagnosis of 48–60 months [73,74]. The most frequent clinical manifestations are pneumonia, otitis media, and diarrhea [73,74]. The prevalence of bronchiectasis in XLA varies by cohort, ranging from 10% to 44% [73,75].

Bronchiectasis is a major cause of morbidity and mortality in XLA, and its presence is associated with a faster decline in FEV_1_ than in CVID [76]. Immunoglobulin replacement is the main treatment; however, many patients continue to have recurrent infections, progressive lung damage, and reduced quality of life despite higher IgG doses [77,78,79]. Patients with XLA and bronchiectasis can continue to show lung disease progression even with adequate IgG replacement therapy [78]. Respiratory failure is one of the most frequent causes of death in these patients [75].

There is a need to investigate the mechanisms regulating inflammation in XLA to improve therapeutic strategies and optimize outcomes.

### 3.5. Other Primary Humoral Immunodeficiencies

Although there is little information, other types of PHI may be associated with bronchiectasis.

#### 3.5.1. Hyper-IgM Syndrome (HIGM)

HIGM is a primary immune disorder in which the patient has normal or elevated IgM levels but very low or absent levels of the other main immunoglobulins (IgG, IgA, and sometimes IgE) [13]. The most common form is X-linked, caused by mutations in the CD40L gene in T lymphocytes, although there are other genetic variants. The defect lies in class-switch recombination, where B lymphocytes switch from producing IgM to other antibody classes. Treatment is based on IgG replacement therapy.

In a cross-sectional study, Moazzami et al. reported that, among 62 patients with HIGM, more than 50% had pneumonia and 14.1% had bronchiectasis [80].

#### 3.5.2. Selective IgE Deficiency

This is a very rare primary humoral immunodeficiency characterized by markedly reduced serum IgE concentrations, with preservation of the levels of the other immunoglobulin isotypes [81]. The available literature on this condition remains scarce. Picado et al., in a retrospective study of 52 adults, observed that a considerable number of patients presented with autoimmune diseases, but also that a significant proportion exhibited an increased frequency and severity of respiratory tract infections, both upper and lower, such as chronic bronchitis (34.6%), pneumonia (30.7%), and even bronchiectasis (30.7%) [82].

#### 3.5.3. Extremely Rare Forms

Other very rare PHI variants include unclassified hypogammaglobulinemia, specific antibody deficiency (SPAD), specific polysaccharide antibody deficiency (SAD) and heavy- and light-chain deficiencies. These conditions have not been associated with bronchiectasis in the literature but are associated with recurrent respiratory infections, which favor the development of bronchiectasis [2].

## 4. Future Directions

The growing recognition of PHI as a relevant etiology of bronchiectasis contrasts sharply with the limited, often heterogeneous evidence base that currently guides clinical practice in this setting. Future research must therefore prioritize robust, prospective characterization of patients with bronchiectasis and PHI, including precise immunological phenotyping, longitudinal assessment of lung function and exacerbation burden, and detailed microbiological profiling. Such work should clarify which specific defects (for example, IgG2 deficiency, combined subclass deficiencies, or CVID) carry the greatest risk of developing bronchiectasis and of progression once established, and at what time-points immunoglobulin replacement and other interventions have the greatest preventive impact.

Regarding new therapeutic approaches, emerging treatments such as dipeptidyl peptidase 1 (DPP1) inhibitors, along with other anti-inflammatory and host-directed strategies, have been tested almost exclusively in populations without overt immunodeficiency [83,84]. The strict exclusion of patients with PHI from most phase II and III bronchiectasis trials leaves a major evidence gap: the efficacy, safety, optimal dosing, and risk–benefit balance of these agents in individuals with underlying antibody defects remain essentially unknown. As a result, clinicians need to extrapolate experiences from non-immunodeficient cohorts or to withhold potentially beneficial therapies from a group with particularly high morbidity. Future clinical trials should explicitly address this limitation by either including carefully phenotyped PHI subgroups with pre-specified stratified analyses, or by designing dedicated studies focused on immunodeficiency-associated bronchiectasis. Pragmatic trials and well-controlled observational studies, nested within large registries, may offer a feasible intermediate step where traditional randomized designs are challenging.

National and international registries will be central to these efforts. Existing immunodeficiency registries (such as ESID and USIDNET) and bronchiectasis registries, such as EMBARC (Europe) and RIBRON (Spain), among others, already capture complementary aspects of these conditions, but are rarely integrated. Linking or harmonizing these datasets would allow a more accurate estimation of the true prevalence of bronchiectasis across PHI subtypes, better description of natural history, and more granular evaluation of outcomes according to immunoglobulin replacement strategy, airway clearance practices, chronic antibiotic use, and exposure to newer therapies such as DPP1 inhibition. Importantly, registries can facilitate post-marketing surveillance of off-label or early-access use of novel bronchiectasis treatments in patients with PHI, providing real-world safety and effectiveness data that are unlikely to be obtained promptly through trials alone.

Furthermore, systematic measurement of serum immunoglobulins and IgG subclasses in all adults with bronchiectasis of unknown cause, as recommended by current guidelines, remains inconsistent in routine practice. Education of respiratory physicians, immunologists, and primary care clinicians to recognize PHI as a “treatable trait” in bronchiectasis, and to refer patients early to specialized centers, is essential. Ultimately, the convergence of biologically informed phenotyping, inclusive trial design, and high-quality registry data offers the most promising route towards genuinely personalized care for patients with bronchiectasis and PHI.

## 5. Conclusions

There is a wide spectrum of primary humoral immunodeficiencies that, to varying degrees, predispose to recurrent respiratory infections and consequently increase the likelihood of developing bronchiectasis (Figure 1). Among these, common variable immunodeficiency—given its combination of multiple humoral and cellular defects—appears to be the condition most strongly associated with bronchiectasis. The question of whether isolated or combined deficiencies can directly lead to bronchiectasis remains under debate; however, current evidence suggests that IgG2 deficiency, in particular, shows a stronger association.

The clinical relevance of selective or combined IgG deficiencies lies in the fact that they represent a treatable trait in bronchiectasis, as highly effective replacement therapies are available. Large-scale studies, leveraging existing international and national registries of primary humoral immunodeficiencies, are needed to clarify the precise relationship between these disorders and the pathogenesis and impact of bronchiectasis. In the meantime, in any patient with bronchiectasis not attributable to other causes and preceded by recurrent respiratory infections, it is essential to rule out quantitative or functional immunoglobulin deficiencies.

## Figures and Tables

**Table 1:** Predominantly antibody deficiencies according to the definition of IUIS (modified from [5]).

Group	Brief Description
1. Severe reduction in all serum immunoglobulin isotypes with profoundly decreased or absent B cells (agammaglobulinemia)	Marked decrease in IgG, IgA and IgM with very low/absent circulating B cells. Typical genetic defects: BTK (X-linked agammaglobulinemia), IGHM (μ heavy chain), IGLL1 (λ5 surrogate light chain), CD79A/CD79B (Igα/Igβ), BLNK, and other genes affecting early B-cell development.
2. Severe reduction in at least 2 serum immunoglobulin isotypes with normal or low number of B cells (CVID phenotype)	Reduction of ≥2 immunoglobulin isotypes (usually IgG with low IgA and/or IgM) with normal or reduced B-cell counts. Includes common variable immunodeficiency (CVID) and monogenic CVID-like disorders (e.g., ICOS, CD19, CD81, CD20/MS4A1, TNFRSF13B/TACI, TNFRSF13C/BAFF-R, and other genes associated with CVID phenotypes).
3. Severe reduction in serum IgG and IgA with normal/elevated IgM and normal number of B cells (Hyper-IgM phenotypes)	Characterized by impaired immunoglobulin class-switch recombination: low/absent IgG and IgA with normal or increased IgM. Classical hyper-IgM syndromes due to defects in CD40LG (CD40L), CD40, AICDA (AID) or UNG, among others.
4. Isotype, light chain, or functional deficiencies with generally normal numbers of B cells	Deficiency of one or more immunoglobulin isotypes or light chains. Includes selective IgA deficiency, IgG subclass deficiency, selective IgM deficiency, isolated heavy chain deficiencies (e.g., α, γ, μ) and light chain deficiencies (κ or λ). Also includes impaired specific antibody responses to vaccines and/or natural infections (especially polysaccharide antigens such as pneumococcal vaccines), as well as transient hypogammaglobulinemia of infancy.

IUIS: International Union of Immunological Societies; Ig: Immunoglobulin; BTK: Bruton’s tyrosine kinase; CVID: Combined variable immunodeficiency.

**Table 2:** Immunoglobulins: function and consequences of deficiency [12,13,14].

Immunoglobulin (Ig)	Main Functions	Subclasses/Characteristics	Main Clinical Consequences of Deficiency
IgA	Defense at mucosal surfaces (respiratory, gastrointestinal, urogenital) Neutralization of pathogens and toxins on mucosa Immune exclusion (prevents adhesion and invasion) Passive protection through breast milk (secretory IgA)	IgA1: Predominant in serum and in respiratory and upper gastrointestinal secretions; efficient in neutralization and agglutination. IgA2: More resistant to bacterial proteases; relatively more abundant in intestinal secretions and colon.	Often asymptomatic. Recurrent sinopulmonary and gastrointestinal infections Higher frequency of allergic diseases Increased risk of some autoimmune diseases (e.g., celiac disease, autoimmune thyroiditis) Risk of anaphylactic reactions to blood products containing IgA in a minority of patients
IgM	First antibody in primary immune response Potent activator of the classical complement pathway Efficient neutralization and agglutination of pathogens (pentameric form) B-cell receptor in its membrane form (monomeric)	No clinically relevant subclasses. Circulates mainly as a pentamer; membrane IgM on naïve B cells is monomeric.	Selective or predominant IgM deficiency is rare and variably expressed: Increased susceptibility to bacterial infections, especially of the respiratory tract Possible association with autoimmune diseases in some patients Some cases show increased frequency of allergic diseases
IgG	Opsonization and neutralization of bacteria, viruses and toxins Activation of classical complement pathway (especially IgG1 and IgG3) Transfer across the placenta (major role in neonatal immunity) Long-term systemic humoral immunity	IgG1: Most abundant subclass; strong response to protein antigens; good opsonization and complement activation; efficiently crosses the placenta. IgG2: Main response to bacterial capsular polysaccharides; weaker placental transfer. IgG3: Very effective complement activator with good opsonizing capacity; responds mainly to protein antigens; shorter half-life. IgG4: Poor activator of complement; often associated with chronic antigen exposure and immunomodulatory/anti-inflammatory responses.	Recurrent and sometimes severe sinopulmonary infections and otitis More pronounced susceptibility when combined with low total IgG. Recurrent infections by encapsulated bacteria Poor responses to polysaccharide vaccines. Recurrent upper and lower respiratory tract infections, often in adults Frequently associated with other subclass deficiencies. Very common and often found in healthy individuals Usually asymptomatic when isolated In some cases, reported in association with atopic or autoimmune conditions, but a causal role is uncertain.

## Data Availability

No new data were created or analyzed in this study.

## Abbreviations

The following abbreviations are used in this manuscript:

PHI	Primary Humoral Immunodeficiency
ESID	European Society for Immunodeficiencies
USIDNET	United States Immunodeficiency Network Registry
IUIS	International Union of Immunological Societies
Ig	Immunoglobulin
CVID	Common Variable Immunodeficiency
COPD	Chronic Obstructive Pulmonary Disease
XLA	X-Linked Agammaglobulinemia
HIGM	Hyper IgM Syndrome
DPP1	Dipeptidyl peptidase 1
